# Diversity of mutated antigen recognition by tumor infiltrating lymphocytes from patients with metastatic melanoma

**DOI:** 10.1186/2051-1426-3-S2-P9

**Published:** 2015-11-04

**Authors:** Jessica S Crystal, Todd D Prickett, Jared J Gartner, Maria R Parkhurst, Yong-Chen Lu, Alena Gros, Yong Li, Kasia Trebska-Mcgowan, Mona El-Gamil, Steven A Rosenberg, Paul F Robbins

**Affiliations:** 1 NCI/NIH, Rutgers Robert Wood Johnson Medical School, New Brunswick, NJ, USA; 2 NCI/NIH, Bethesda, MD, USA; 3 NIH/National Cancer Institute, Bethesda, MD, USA; 4 Surgery Branch / National Cancer Institute / National Institutes of Health, Bethesda, MD, USA; 5 NIH/NCI, Bethesda, MD, USA; 6 Surgery Branch/National Cancer Institute / National Institutes of Health, Bethesda, MD, USA

## Introduction

The adoptive cell transfer (ACT) of autologous tumor infiltrating lymphocytes (TIL) can mediate durable, complete tumor regression in approximately 20% of patients with metastatic melanoma. Recent observations suggest that autologous melanoma TIL administered to multiple patients in ACT protocols can recognize 1 or more tumor-specific somatic mutations, findings facilitated by recent advances in whole exome sequencing and RNA-seq methods.

## Methods

In an attempt to evaluate the antigenic diversity of TIL and gain some insights into the potential association between the recognition of mutated antigens and clinical responses to TIL therapy, we analyzed between 5 and 30 individual cultures derived from resected melanoma tumor fragments and pooled populations of administered TIL from each patient for their ability to recognize mutated antigens expressed by patients' tumors. Samples from 11 patients evaluated in this initial study include 5 who exhibited durable complete tumor regressions, 1 who exhibited a partial response, and 5 who were non-responders to ACT. Screening assays were carried out by evaluating the interferon gamma release stimulated by the co-culture of patient TIL with autologous dendritic cells or EBV transformed B cells that were transfected with up to sixty two tandem mini-genes encoding mutated antigens identified by sequencing patient tumors.

## Results

Using this approach, we were able to identify between 2 to 11 mutated antigens per patient that were targeted by TIL in 9 of the 11 patients. The TIL generated from 2 of the 5 patients who did not respond to ACT failed to recognize any mutated antigens tested, but recognized an autologous tumor cell line. For each of the 9 patients demonstrating mutated antigen reactivity, at least 1 immuno-dominant mutated antigen was recognized by the majority of the evaluated TIL fragment cultures and the bulk infusion TIL, and 1 or more sub-dominant mutated antigens recognized by one or a relatively small percentage of the screened TIL fragment cultures could be identified. Future studies will be directed at developing approaches to tumor immunotherapy based upon the identification and isolation of T cells reactive with mutated epitopes.

